# Everything You Always Wanted to Know about Sexes

**DOI:** 10.1371/journal.pbio.0020183

**Published:** 2004-06-15

**Authors:** John Whitfield

## Abstract

What defines a sex? Although we tend to think there are only two - males and females - there are many different ways to mix and match the attributes of sexes

From a human perspective, sexes seem a relatively simple thing to get one's head around—there are females, and there are males. But our perspective seems biased and narrow when applied to life as a whole, says evolutionary biologist Laurence Hurst of the University of Bath, United Kingdom.“If you were a single-celled alga sitting in a pond, you wouldn't see the world as splitting into males and females.”

In fact, different species have evolved a bewildering number of ways to mix and match the attributes of sexes. Some do not have males and females, but have adaptations that mean each individual performs a specific role during sex. There are other species of which every member is sexually equivalent, but individuals nevertheless divide into groups for the purposes of mating. And in some species, individuals make both eggs and sperm (Box 1). This biological diversity has produced a semantic muddle among biologists—everyone who thinks about the evolution of sexes seems to have a slightly different take on what a sex is. “The literature is highly confusing—we need to clarify our terminology,” comments Rolf Hoekstra, a geneticist at the University of Waageningen in the Netherlands.

As things stand, there are three main aspects to the definition of a sex: who you are, who you can mate with, and who your parents are. The third part of this trinity—parental number—shows the least variation in nature. No known organism needs more than one mother and one father. But even this assumption is now starting to break down at the level of biological systems. In a recently discovered hybrid system within the harvester ant genus Pogonomyrmex, queens must mate with two types of males to produce both reproductive individuals and workers (Figure 3). These ants are the first species known which truly has more than two sexes—with colonies effectively having three parents— argues Joel Parker of the University of Lausanne, Switzerland.

**Figure 3:**
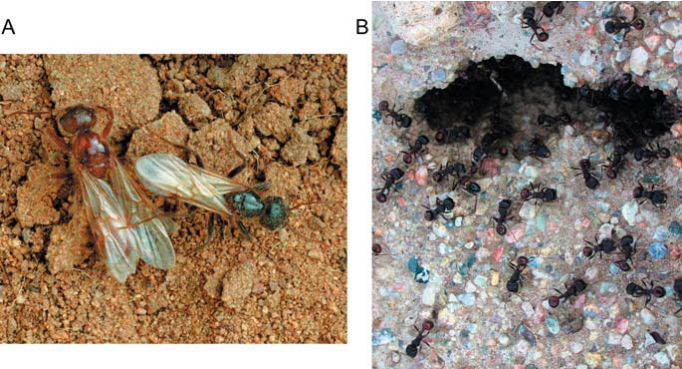
An Ant with Three Sexes? (A) Two males from the harvester ant genus Pogonomyrmex, one from each genetic strain. In a recently discovered hybrid system, queens must mate with both types of males to produce reproductives and workers. Photo courtesy of Charles Hedgcock, Charles Hedgcock Photography, Tucson, Arizona, United States. (B) Hybrid workers emerging from a nest. Photo courtesy of Veronica Volny, University of California, Berkeley, California, United States.

Parker's ideas might reactivate evolutionary biologists' interest in sexes, which has lain somewhat dormant since the 1990s. It could also provide a new route to experiments— something often lacking in the field. Not everyone agrees that it makes sense to define the ants' genetic quirks as new sexes. Each ant is still only a mix the genes from no more than two parents, after all. But Parker believes that our current ideas about mating systems may not be adequate to describe the ingenuity of evolution. “Until you see a three-sex system, you don't know what it'll look like,” he says.

## Little and Large

To address whether these ants have more than two sexes, we first need to look at other candidates for sexes, their numbers in different species, and how these systems evolved. One thing biologists do agree on is that males and females count as different sexes. And they also agree that the main difference between the two is gamete size: males make lots of small gametes—sperm in animals, pollen in plants—and females produce a few big eggs. But researchers also think that before males and females evolved, sex occurred between organisms with equal-sized gametes, a state called isogamy.

Evolutionarily speaking, an isogamous species faces two pressures. Individuals can make more smaller gametes, thus increasing their potential number of offspring, or they can make fewer bigger gametes, thus giving their offspring a better start in life by providing them with more resources. Theoretical analyses suggest that this pressure is particularly great if being big carries large benefits, making isogamy unstable. The original identical gametes will evolve towards the opposite ends of the size spectrum.

**Figure 4:**
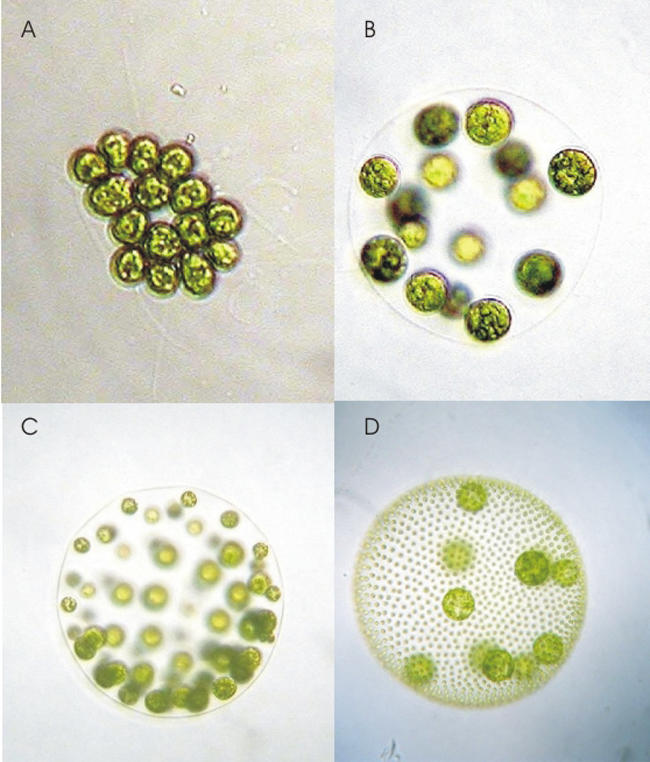
Four Different Species of Volvocales Algae (A) Gonium pectorale, (B) Eudorina elegans, (C) Pleodorina californica, and (D) Volvox carteri. These are unicellular organisms that live in colonies and have both large and small gametes. Photo courtesy of Aurora M. Nedelcu, from the Volvocales Information Project (http:\\www.unbf.ca\vip\index.htm).

In many species, however, one size of gamete still fits all. The organisms that have hung on to isogamy are found among the less complex branches of life, such as fungi, algae, and protozoa. This might be because large gametes, yielding well-funded zygotes, are likely to be more strongly selected if the resulting offspring needs to grow into a large and complex organism. The benefits of large gametes in simple and unicellular organisms are not so obvious. Some support for this hypothesis comes from the algae belonging to the group Volvocales. The variation in gamete size within each species matches its degree of complexity. For example, the unicellular species Chlamydomonas rheinhardtii is isogamous, while Volvox rouseletti, which lives in balls of up to 50,000 cells, has large and small gametes (Figure 4).

## The Opposite of Sexes?

The question of sexes, and their number, is complex in isogamous species. Such species still typically comprise different groups for mating purposes. They have genes that allow them to mate with everyone except those belonging to the same “mating type” (this is presumably to avoid inbreeding and to produce offspring that are genetically diverse to cope with environmental change or biological enemies). Species with mating types, rather than males and females, aren't limited to two interbreeding groups: the ciliate protozoan Tetrahymena thermophila has seven, and the mushroom Schizophyllum commune has more than 28,000, for example. Some biologists call these mating types sexes; others think that, in the absence of traits other than sexual compatibility or the lack thereof, it makes more sense to view species with many mating types as having no sexes, rather than lots.

Yet most isogamous species have only two mating types. This seems perverse—it excludes half the population as potential mates without gaining the benefits of specialization in sexual biology. With William Hamilton, Hurst came up an explanation for this apparent inefficiency.

Two-group mating systems, they proposed, evolved as a way for genes in the nucleus to police the DNA in organelles. Cellular structures with their own genomes, such as mitochondria and chloroplasts, can divide more rapidly than the cells that house them. If the inheritance of organelles was biparental, selfish mutations in their DNA could spread rapidly, Hurst and Hamilton showed. A nuclear gene that enforces uniparental inheritance of organelles, along with a label that allows such cells to recognize each other so that their nuclear genes can share the benefits of cytoplasmic policing, should be favored.

The mating biology of isogamous species offers considerable support for this idea. The aforementioned C. rheinhardtii, for example, comes in two mating types called plus and minus. When the two fuse, the plastid of the minus cell is detroyed. Most isogamous species that fuse cells have a similar mechanism. Male-killer parasites such as Wolbachia, a parasite of arthropods, show the selection pressure that intracellular passengers can exert (see also the primer by Wernegreen in the March issue of *PLoS Biology*). And cellfusion experiments hint that biparental inheritance of organelles does indeed cause problems, says Hurst. “Hybrids are often rubbish, but they can be better if a drug is administered that inhibits the mitochondria of one cell line.”

The species that have lots of mating types, such as ciliate protozoa, exchange nuclear DNA, but not cytoplasm, and hence not intracellular organelles. Since individuals are freed from the need to police their organelles or keep out parasites, selection favors the widest assortment of possible mates, and thus the evolution of a large number of mating types so that one's own type—which one can't mate with—is a small subset of the population. It is possible to imagine species with cytoplasmic policing likewise having many mating types, but such a situation would be much more prone to break down and be invaded by selfish agents than one with two clearly defined types, which is what we usually see in nature. Some have argued that cytoplasmic policing might also be a selective force for different-sized gametes. Sperm could be small so that they do not import mitochondria into the egg.

More than a decade after he devised it, Hurst's is still the leading hypothesis explaining the number of mating types in a species. But experimental evidence remains frustratingly elusive. “I wouldn't say I was entirely satisfied,” says Hurst. “We've got all these ideas, and they turn out to be quite hard to test—there's no simple thing one can do on a single species.” There are species where the uniparental inheritance of organelles is not so strictly enforced, says Hoekstra, such as yeasts and plants. “It's not easy to see if selection [on organelles] is strong enough,” he says.

## Three's Company

Yet even in a species such as S. commune, with its thousands of mating types, each sexual encounter involves only two cells. Nor are we likely to find a species that defies this pattern. The technical difficulties of combining more than two sets of genetic information into one individual, and of parceling out that information during meiosis, must be vast, says Brian Charlesworth of the University of Edinburgh. “We've reached the point of two cells fusing, and stuck with that; two cells are probably just as good as three,” he says.

The ant colonies that Parker suggests have three parents are a hybrid of the species Pogonomyrmex rugosus and P. barbatus. The hybrids have not yet been classed as a new species, but they are well established across the southwestern United States, and there is no evidence of contemporary gene flow between hybrids and their parent species.

Each ant has one parent if it is male, because male ants are produced from unfertilized eggs, or two if it is female. But each sex also comes in two genetic strains. If a queen mates with a male of her own strain, her offspring will be queens, and if she mates with a male from the other strain, the sperm will give rise to workers. So, for a colony to function fully it—and the queens it produces, because workers raise queens—must have two fathers and one mother. And if any one group were to disappear, the population as a whole would go extinct—unlike fungal mating types, where it's easy to imagine that the species would carry on if a few disappeared. “If you lose any one, the whole thing collapses,” says Parker. “It's really different from any other system.”

So, Parker argues, Pogonomyrmex has four sexes: the males and females of each strain. The idea is particularly potent if one views a social insect colony as a “superorganism,” with the workers equivalent to the cells of a body. It's as if a female mates with one male to produce her offspring's somatic cells, and another to produce its germ cells. The ants form chaotic mating swarms, so most queens have no problem mating multiply and getting sperm from males of both strains, although one would expect that males would strongly favor mating with females of their own strain.

It's not known how the system originated. Separating the worker and reproductive castes by genetics—other social insects do this by environment, that is, by rearing workers and reproductives differently—may allow selection to operate more efficiently on each lineage, and the workers may benefit from hybrid vigor: field researchers report them as being highly aggressive. In an echo of Hurst's hypothesis, the system also mixes mitochondrial and nuclear genes differently in queens and workers.

Some evolutionary biologists, such as Charlesworth, do not consider Pogonomyrmex'*s* mating types sexes, arguing that to define sexes in yet another way only confuses the picture further. “[The ants] are an interesting system, but I wasn't persuaded by Parker's interpretation,” Charlesworth says. “I'm not a fan of the idea that it's useful to use the word ‘sex’ to describe compatibility between mating types—it muddies the waters.” Others are more positive towards Parker's interpretation: “It deserves to be taken seriously,” says evolutionary biologist Eörs Szathmáry of the Collegium Budapest in Hungary. “He's thrown a stone in the water—now we need to see what kinds of ripples it makes. You can't falsify a definition in the way you can a hypothesis; what determines their fate is whether people find them useful or not.”

Species in which some individuals give up their reproductive opportunities to form part of a breeding group, such as slime molds, might have a system similar to that of the ants, Parker believes. “There may be hidden mating incompatibilities,” he says. “Now [that] people know to look, we're going to start seeing more of these systems.”

## 

**Figure 1:**
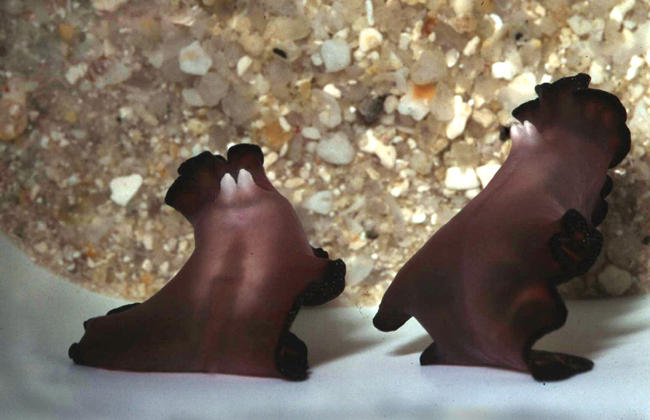
Two Individuals of Pseudobiceros bedfordi About to Have a Sperm Battle Species of the flatworm genus Pseudobiceros are hermaphroditic and have two penises that are used to inject sperm into the partner. P. bedfordi is exceptional in that it applies sperm onto the partner's skin rather than injecting it. Photo courtesy of Nico Michiels.

**Figure 2:**
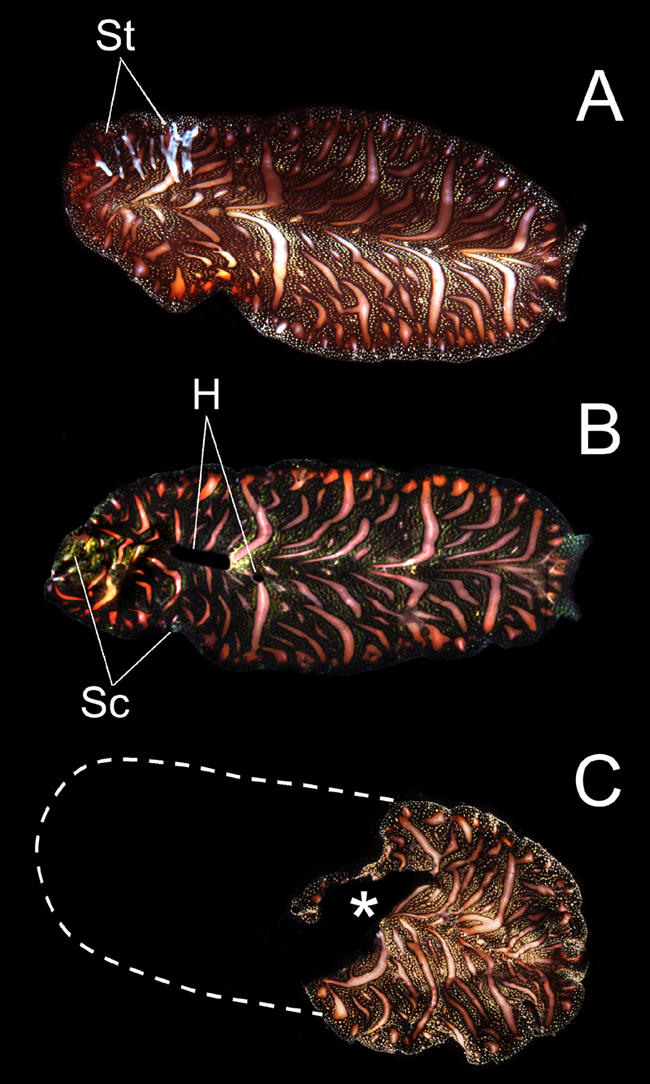
Scars of Sex (A) Streaks of sperm (St) received after a mating interaction in the hermaphroditic flatworm, Pseudobiceros bedfordi. (B) Received sperm appears to “burn” holes (H) in the receiver. Some (unknown) component of the ejaculate dissolves the skin tissue. Sc, scar tissue. (C) Exceptional case where an individual received a large amount of sperm somewhere in the middle of the body, resulting in a large hole (asterisk). The the body subsequently tore in two. Individuals like these are occasionally found in the field and can regenerate much of their body. Photo courtesy of Nico Michiels.

**Box 1. The Best of Both Worlds?**

One option for dividing up the sexes is “both”—hermaphroditism. This might seem like an ideal solution—everyone becomes a potential partner, and everyone can bear offspring. In practice, however, hermaphroditism is uncommon among multicellular animals. The reasons are similar to those explaining why evolution favors unequal-sized gametes—once sexes have evolved, it's better to commit all one's resources to one role or the other, rather than try and be a jack-of-all-trades. After all, there are many good uses for mating resources other than simply producing eggs or sperm. An animal could defend a territory or provide parental care, for example.

Hermaphroditism, however, is useful if one's sexual options are severely limited. In particular, it can be favored when encounters with potential mates are extremely rare. It makes no sense for an animal to invest heavily in the biological equipment of maleness, say, if it will have almost no opportunities to use it: better to hedge your bets. Animals with low or unpredictable population densities and those that are immobile, have poor senses, or lack long-distance signalling are often hermaphroditic. These include sponges, worms—whether flat, nematode, or annelid—and many molluscs (and, of course, plants, the majority of which are hermaphroditic). Most hermaphrodites still need to find at least one mate in their lifetimes: the cost of inbreeding prevents self fertilization from becoming common.

Hermaphroditic animals have some weird sexual adaptations. Helix aspersa snails shoot calcareous love darts into one another. And when the marine flatworm Pseudobiceros bedfordi mates, each worm has two penises, which they fence with in a battle to smear one another with sperm without being fertilized themselves in the process (Figures 1 and 2).

Such oddities result when the mating opportunities of a hermaphroditic species increase, and specialization starts to become more favorable, says evolutionary biologist Nico Michiels, of the University of Muenster in Germany. In a species with two separate sexes, males and females often have different ideas about whether a mating is a good idea—males tend to be keener, females tend to be choosier. The result can be an evolutionary arms race, with each sex evolving adaptations that help them get their way. By exercising mate choice, each sex has some influence on the types of adaptations that evolve—anything too outlandish is unlikely to be favored. This counterbalance is not present in hermaphrodites, however. Rather than having one half of a species resist a particular mating strategy, the whole species is just as likely to adopt it. “Hermaphrodites run into awkward and bizarre mating conflicts,” says Michiels.

Michiels believes that hermaphroditism was the ancestral state for animals, and thinks that we might be able to find the relics of this past in contemporary species with separate sexes. To test these ideas, he is searching for groups containing closely related hermaphroditic and bisexual species. Such taxa are very rare, however.
